# Supplementary material for: Design and Fabrication of Sodium Alginate/Carboxymethyl Cellulose Sodium Blend Hydrogel for Artificial Skin
Source: Gels. 2021 Aug 9;7(3):115. doi: 10.3390/gels7030115 (PMC8395816; doi:10.3390/gels7030115)
Supplement: Supplementary file 1 [file gels-07-00115-s001.zip › gels-1322804-supplementary.pdf]

gels

Design and Fabrication of Sodium Alginate/Carboxymethyl Cellulose Sodium Blend Hydrogel for Artificial Skin

Kun Zhang [1,2], Yanen Wang [1,2,*,†], Qinghua Wei [1,2,*], Xinpei Li [1,2], Ying Guo [1,2] and Shan Zhang [1,2]

[1] Industry Engineering Department, School of Mechanical Engineering, Northwestern Polytechnical University, Xi'an 710072, China; npu_zk@sina.com (K.Z.); lixinpei@mail.nwpu.edu.cn (X.L.); guoying0402@sina.com (Y.G.); zhangshanzs33@163.com (S.Z.)
[2] Institute of Medical Research, Northwestern Polytechnical University, Xi'an 710072, China
* Correspondence: wangyanen@126.com (Y.W.); weiqinghua@nwpu.edu.cn (Q.W.)
† These authors contributed equally to this work and should be considered co-first authors.

Supplementary Materials

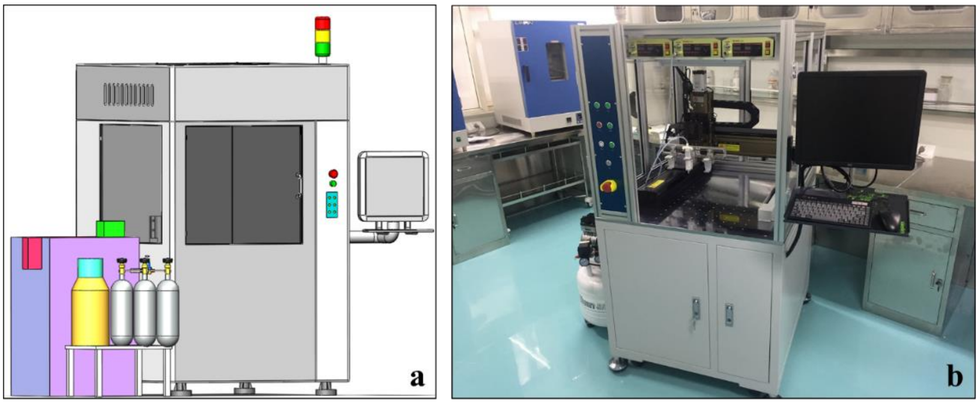

Figure S1. (**a**) Bio-printer control cabinet structure diagram; (**b**) Bio-printer pneumatic extrusion system

Citation: Kun, Z.; Yanen, W.; Qinghua, W; Ying, G; Shan, Z. Design and Fabrication of Sodium Alginate/Carboxymethyl Cellulose Sodium Blending Hydrogel for Artificial Skin. *Gels* **2021**, *7*, 115. https://doi.org/10.3390/gels7030115

Academic Editor: David Díaz Díaz

Received: 16 July 2021
Accepted: 6 August 2021
Published: 9 August 2021

Publisher's Note: MDPI stays neutral with regard to jurisdictional claims in published maps and institutional affiliations.

Copyright: © 2021 by the authors. Licensee MDPI, Basel, Switzerland. This article is an open access article distributed under the terms and conditions of the Creative Commons Attribution (CC BY) license (http://creativecommons.org/licenses/by/4.0/).
